# Vibration-Based Anomaly Detection for Induction Motors Using Machine Learning

**DOI:** 10.3390/s25030773

**Published:** 2025-01-27

**Authors:** Ihsan Ullah, Nabeel Khan, Sufyan Ali Memon, Wan-Gu Kim, Jawad Saleem, Sajjad Manzoor

**Affiliations:** 1 Department of Electrical Engineering, COMSATS University Islamabad Abbottabad Campus, Abbottabad 22060, Pakistan; ihsan@cuiatd.edu.pk (I.U.); nabeeltasir@gmail.com (N.K.); jawadsaleem@cuiatd.edu.pk (J.S.); 2 Department of Defense Systems Engineering, Sejong University, Gwangjin-gu, Seoul 05006, Republic of Korea; 3 Marine Domain & Security Research Department, Korea Institute of Ocean Science & Technology, Busan 49111, Republic of Korea; kimwangu@kiost.ac.kr; 4 Department of Electrical Engineering, Mirpur University of Science and Technology, Mirpur AJK 10250, Pakistan; sajjad.ee@must.edu.pk

**Keywords:** deep neural networks, fault detection, frequency domain analysis, K-nearest neighbors, statistical feature, support vector machines, time domain analysis, vibration monitoring

## Abstract

Predictive maintenance of induction motors continues to be a significant challenge in ensuring industrial reliability and minimizing downtime. In this study, machine learning techniques are utilized to enhance fault diagnosis through the use of the Machinery Fault Database (MAFAULDA). A detailed extraction of statistical features was performed on multivariate time-series data to capture essential patterns that could indicate potential faults. Three machine learning algorithms—deep neural networks (DNNs), support vector machines (SVMs), and K-nearest neighbors (KNNs)—were applied to the dataset. Optimization strategies were carefully implemented along with oversampling techniques to improve model performance and handle imbalanced data. The results achieved through these models are highly promising. The SVM model demonstrated an accuracy of 95.4%, while KNN achieved an accuracy of 92.8%. Notably, the combination of deep neural networks with fast Fourier transform (FFT)-based autocorrelation features produced the highest performance, reaching an impressive accuracy of 99.7%. These results provide a novel approach to machine learning techniques in enhancing operational health and predictive maintenance of induction motor systems.

## 1. Introduction

Industrial systems across manufacturing, energy, and transportation sectors are powered by induction motors [1]. Operational efficiency is directly impacted by their reliability, where significant economic losses, production delays, and potential safety hazards can be caused by unexpected failures. While predictive maintenance has been established as a crucial strategy in mitigating these challenges, limitations have been found in traditional diagnostic methods that rely on manual inspection and periodic maintenance. Through recent advances in machine learning and signal processing, this landscape has been transformed and more accurate, proactive approaches to equipment monitoring have been enabled [2].

Catastrophic failures can occur when faults in induction motors, arising from complex interactions between mechanical and electrical components, are left undetected [3,4]. Vibration and acoustic emission signals have been identified as essential indicators for fault detection, through which statistical techniques and probabilistic models have been widely adopted over recent decades [5,6]. Intelligent solutions for predictive maintenance in rotating machinery have been implemented by manufacturers, particularly where remote monitoring applications are concerned [7]. Vibration-based condition monitoring has been established as a cornerstone, through which potential faults can be identified by tracking vibration pattern variations [8].

Fault detection strategies have been fundamentally reshaped by data-driven methodologies, where artificial intelligence and advanced signal processing are incorporated [9]. Among the various failure modes by which motor performance can be compromised, imbalance has been identified as a uniquely complex challenge for which sophisticated diagnostic approaches are required. Unlike simpler mechanical failures, the imbalance can be manifested through multiple configurations—mass imbalance, geometric imbalance, and couple imbalance—by which distinct detection challenges are presented and motor reliability and operational efficiency are significantly impacted.

Fault detection reliability is challenged by several obstacles: high-frequency signal noise, dynamic operating conditions, and complex signal propagation mechanisms. In variable industrial environments, where significant signal interference and operational variability are introduced, conventional approaches have been found particularly challenging [10]. By computational constraints, imbalance fault diagnosis is further complicated, especially in real-time processing scenarios where immediate results must be delivered without system performance being compromised. Advanced signal-processing techniques have been developed through recent research, by which precise fault indicators can be extracted under diverse operational conditions [11]. Traditional limitations have been overcome by combining fast Fourier transform (FFT), wavelet transforms, and machine learning algorithms.

Feature extraction has been identified as a critical challenge in imbalance fault analysis. Through hybrid methodologies developed by researchers, time-domain and frequency-domain transformations have been integrated to capture subtle mechanical irregularities [12]. Generalizable features have been emphasized, by which consistency can be maintained across different motor configurations and operating conditions. Detection precision has been enhanced through supervised learning algorithms, ensemble classification methods, and adaptive neural network architectures [13].

The imbalance fault detection field has been continuously advanced through machine learning applications. Particular promise in handling complex motor imbalance characteristics has been demonstrated by deep learning and ensemble methods [14]. By these approaches, fundamental limitations in existing diagnostic methods have been addressed and more robust, adaptable detection models have been developed through which generalization across varied motor configurations and operating conditions can be achieved.

The potential of artificial intelligence in motor fault diagnosis has been highlighted by recent studies [15,16,17]. In our research, deep neural networks (DNNs), support vector machines (SVMs), and K-nearest neighbors (KNNs) have been explored to address the binary classification between “normal” and “imbalance” conditions. In previous work, SVM was combined with long short-term memory (LSTM) networks [18], although a deep understanding of fault-specific patterns was required. Statistical features from vibration and current signals have been employed with SVM in other studies [19], while KNN has demonstrated effectiveness in diagnosing faults in rotating machinery [20,21].

In this work, a novel approach has been developed where traditional and advanced machine learning models are combined for imbalance fault detection. SVM with time-domain features has been employed for binary classification, by which its effectiveness with simpler feature sets can be leveraged, while DNN with FFT-based autocorrelation features has been applied to capture complex fault signatures in the frequency domain. The frequency domain analysis is performed using autocorrelation computed through FFT-based convolution, where the signal is convolved with its time-reversed version. This approach leverages FFT’s computational efficiency for calculating autocorrelation, enabling effective capture of periodic patterns and temporal dependencies in the vibration signals. Using the MAFAULDA dataset [22] and random oversampling techniques [23], the following significant improvements were achieved: SVM accuracy increased from 85.9% to 95.4%; KNN achieved 92.8% accuracy; and our DNN implementation with FFT-based features reached an accuracy of 99.7%. These results align with research where frequency-domain advantages in fault detection have been highlighted [24,25,26].

**Figure 1:**
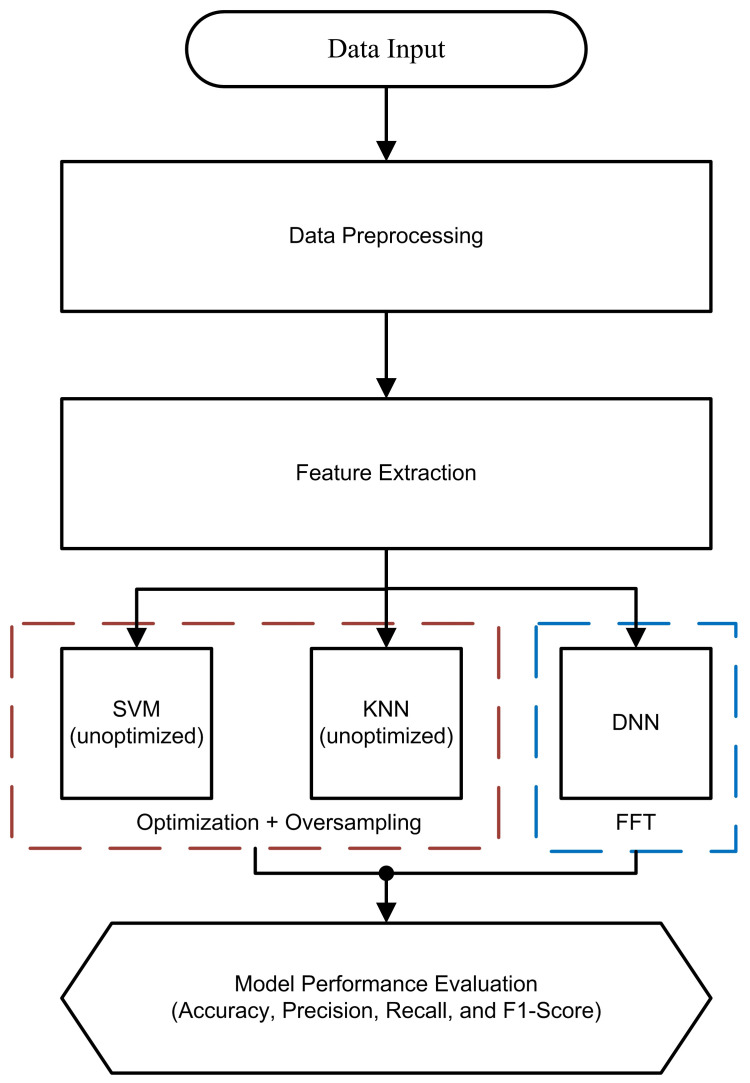
The flowchart of the proposed work: red-dashed box implies the implementation of optimization and oversampling using SVM and KNN; blue-dashed box implies the FFT-based feature extraction applied on DNN.

In this paper, existing research has been extended through the practical application of AI in predictive maintenance. The focus is on vibration-based datasets, preprocessing data/features, and specific fault conditions. Through the MAFAULDA dataset’s alignment with studied fault conditions, both relevance and accuracy in our findings are ensured. Figure 1 illustrates the flowchart of the proposed methods.

The remainder of this paper is organized as follows: In Section 2, the experimental setup, dataset characteristics, and methods are presented, including details of the machinery fault simulator and data acquisition system. Section 3 describes the feature extraction process and the statistical parameters used for fault detection. The feature signal analysis approach and its implementation are detailed in Section 4. Section 5 presents the classification methodology, including the implementation of SVM, KNN, and DNN algorithms along with a discussion on practical considerations and performance evaluations of these algorithms. The experimental results and detailed analysis of each classifier’s performance are presented in Section 6. Section 7 concludes the paper with key findings and suggestions for future research.

## 2. Materials and Methods

### 2.1. Experimental Dataset

**Figure 2:**
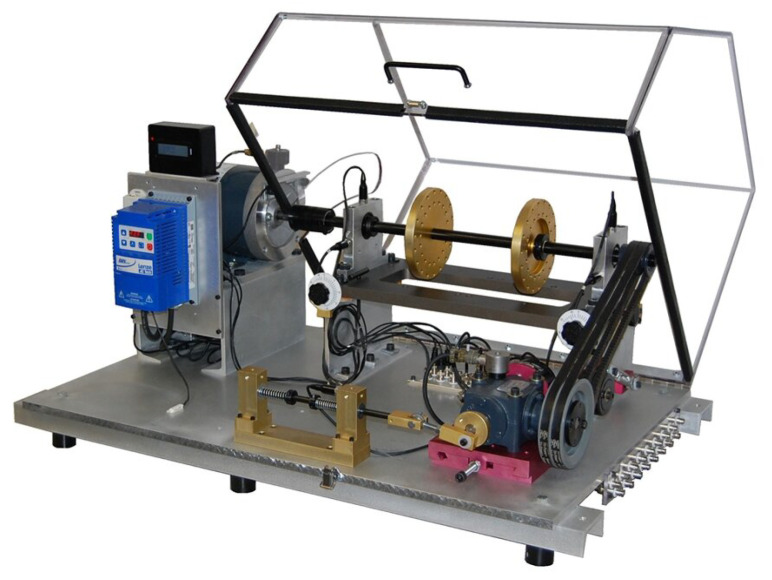
The SpectraQuest machine fault simulator.

The performances of the proposed prediction models were evaluated using the MAFAULDA dataset, which simulates undesirable failure scenarios in rotating machinery, including misalignment, unbalance, and bearing failures. This dataset was collected by the Signals, Multimedia, and Telecommunication Laboratory using the Machine Fault Simulator (MFS), also known as the SpectraQuest Alignment/Balance Vibration Trainer [27], as shown in Figure 2. Although an imbalanced dataset, the MAFAULDA database is specifically designed to assess the performance of diagnostic methods under various operating conditions and fault scenarios. Table 1 provides the default specifications of the MFS trainer [28], which was used to generate the data for this study.

### 2.2. Receiver Operating Characteristic (ROC) Analysis

The receiver operating characteristic (ROC) analysis is a graphical representation of classifier performances across different classification thresholds, enabling a comprehensive model assessment. In addition, the area under the curve (AUC) analysis is used to summarize overall classifier performance. It ranges from 0 to 1, with the following interpretations: 0.5 represents no discriminative capability, 0.5–0.7 represents poor discrimination, 0.7–0.8 represents good discrimination, 0.8–0.9 represents excellent discrimination and values greater than 0.9 indicate outstanding performance. The ROC illustrates the true positive rate (TPR) and false positive rate (FPR), which evaluate the model’s ability to correctly identify normal and faulty conditions.

### 2.3. Methodological Approach to Fault Classification

The comprehensive fault classification methodology employed three advanced machine learning algorithms: SVM, KNN, and DNN. The random classifier, which serves as a baseline predictor by assigning data points to classes with equal probability, is assumed to be a reference model for SVM and KNN classifiers. Each algorithm is systematically evaluated using a robust set of performance metrics such as accuracy, precision, recall, and F1 score to ensure a comprehensive analysis of fault detection capabilities.

#### 2.3.1. Support Vector Machines (SVMs)

Support vector machines represent a powerful machine learning approach that is particularly effective for complex, high-dimensional datasets [29]. The SVM algorithm can handle both linear and non-linear classifications by optimizing the hyperplane separation between different classes. For normalizing the input features during preprocessing, the data are transformed to zero mean $\bar{x}$ and unit variance σ using Equation (1):

(1) $$X_{\mathrm{scaled}}=\frac{X-\bar{x}}{\sigma }$$

**Figure 3:**
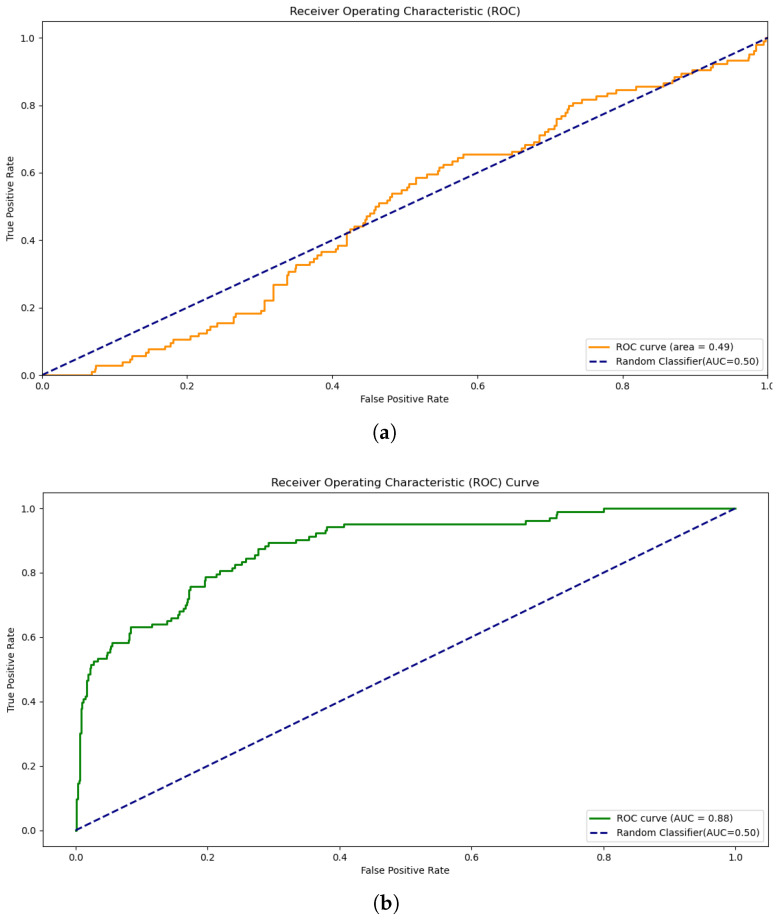

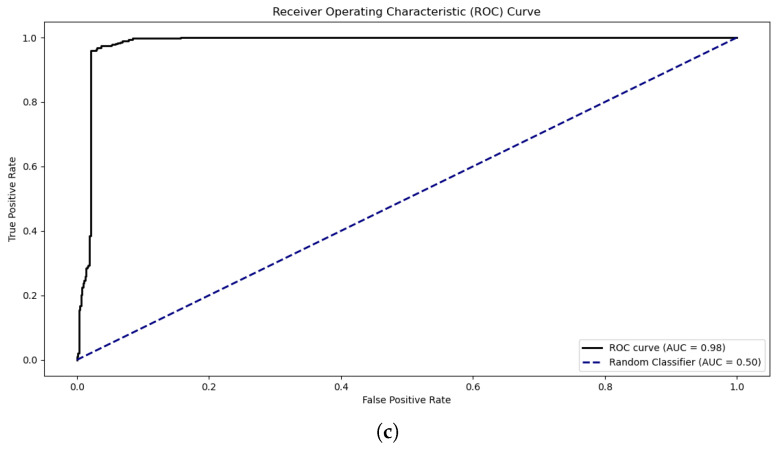
Receiver operating characteristic (ROC) curves of SVM. (**a**) Linear SVM ROC. (**b**) Optimized SVM ROC. (**c**) Optimized SVM using oversampling ROC.

The SVM optimization process balances decision boundary smoothness with training point classification precision. The hyperparameters *C* and γ, detailed in Table 2, are carefully tuned to optimize model performance using Equation (2). The SVM implementation explored three key configurations: unoptimized linear SVM (Figure 3a), which represents the initial baseline performance; optimized SVM with RBF kernel (Figure 3b), which shows significant performance improvement; and oversampled optimized SVM (Figure 3c), which demonstrates the highest discriminative capability.

(2) $$\begin{array}{c} \mathrm{SVM}\left(X_{\mathrm{scaled}},C,\gamma \right)=\arg\min_{w,b,\xi }\left(C\sum_{i=1}^{n}\xi_{i}+\frac{1}{2}{∥w∥}^{2}\right)\\ +\sum_{i=1}^{n}\beta_{i}\xi_{i}+\sum_{i=1}^{n}\alpha_{i}\left(1-y_{i}\left(w\cdot x_{i}+b\right)+\xi_{i}\right), \end{array}$$

where $\xi_{i}$ denotes a slack variable that measures the degree of misclassification, *C* denotes the regularization parameter that controls the trade-off between achieving a low error on the training data and minimizing the model complexity for better generalization, *w* is a weight vector of the hyperplane, *b* denotes a bias of the hyperplane, ∥w∥ represents the squared norm of *w* that controls the margin size (the distance between the separating a hyperplane and the support vectors), $\alpha_{i}$ and $\beta_{i}$ are dual coefficients associated with the constraints that depend on the Lagrange multipliers in the optimization formulation, $y_{i}$ denotes the class label of the ith training sample, $x_{i}$ denotes an input feature of the ith training sample in the feature dataset *X* and *n* represents the total number of training samples.

Figure 3a shows that the area under the ROC is below the random classifier, which indicates that the classifier is not performing well. Figure 3b shows the ROC curve of optimized SVM, which improves the accuracy of both training and testing. Thus, the area under the ROC becomes AUC=0.88 as compared to that of random classifier AUC=0.50, which illustrates excellent discrimination. Figure 3c shows the ROC curve of an oversampled optimized SVM, which achieves an overall accuracy of 95.4% on testing (Table 5, see Section 5). This also demonstrates the model’s high predictive capability. The ROC curve of the classifier illustrates that the model achieved an AUC of 0.98, which indicates outstanding discriminative ability between the normal and imbalanced classes.

#### 2.3.2. K-Nearest Neighbors (KNNs)

**Figure 4:**
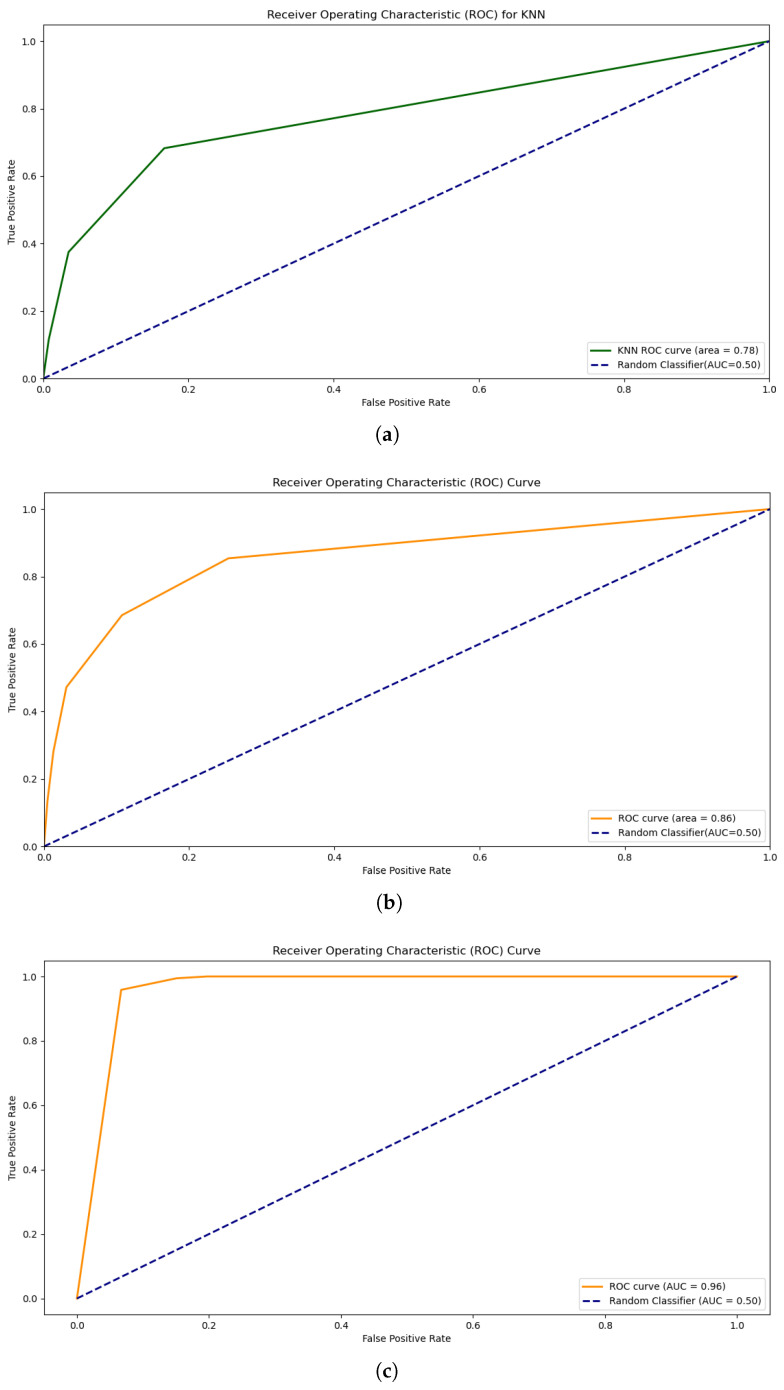
Receiver operating characteristic (ROC) curves of KNN. (**a**) Unoptimized KNN ROC, (**b**) Optimized KNN ROC, (**c**) Oversampled Optimized KNN ROC.

The K-nearest neighbors algorithm represents an instance-based learning technique for classifying unknown data points [30,31]. The KNN algorithm determines class membership based on the majority vote of the k-nearest neighbors. We explored the following two primary distance metrics [32]: Manhattan distance (L1 norm), given by $p1\left(x_{1},x_{2}\right)=\sum_{i=1}^{n}\left|x_{i}-y_{i}\right|$, and Euclidean distance (L2 norm), given by $p2\left(x_{1},x_{2}\right)=\sqrt{\sum_{i=1}^{n}{\left(x_{i}-y_{i}\right)}^{2}}$. The hyperparameters were systematically explored using grid search, as outlined in Table 3. The KNN approach was evaluated using multiple configurations, namely, unoptimized KNN (Figure 4a), which served as the initial performance assessment; optimized KNN (Figure 4b), which showed improved classification accuracy; and oversampled optimized KNN (Figure 4c), which provided enhanced discriminative power.

The ROC curve of unoptimized KNN is shown in Figure 4a, indicating a slightly low AUC of 0.78, which shows that the model is not good enough to predict good results. The ROC of optimized KNN shows AUC=0.86, which is a good discrimination result, and better than the unoptimized KNN, as depicted in Figure 4b. The optimized KNN achieved an accuracy of 89.8% on the test data and approximately 95% on the training data in the confusion matrix, as depicted in Figure 15b and discussed in Section 6.

The over-sampled optimized KNN has AUC=0.96 as shown in Figure 4c, which indicates the outstanding performance of the model, and its overall accuracy is 92.8% on the testing data. This ROC-AUC score level implies that the model has a high level of accuracy in differentiating the normal and imbalanced (faulty) classes.

#### 2.3.3. Deep Neural Networks (DNNs)

**Figure 5:**
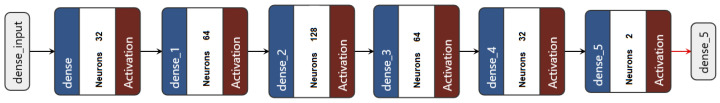
Deep neural network architecture.

We used the dense neural network algorithm [33] to develop the DNN architecture, as shown in Figure 5, for a novel fault classification. The network was fed with autocorrelation features computed through FFT-based convolution. The FFT-based autocorrelation choice for feature extraction offers several key advantages in fault detection. Through autocorrelation analysis, temporal patterns in vibration signals can be effectively captured even when these patterns are obscured by noise in the time domain. This approach is particularly powerful for machinery fault detection, as recurring patterns in the signal are emphasized, making fault signatures more distinguishable. By correlating the signal with itself at different time lags, this method helps isolate fault-specific patterns from the complex vibration signatures typical of induction motors, where multiple mechanical components operate simultaneously. The FFT-based implementation of autocorrelation provides computational efficiency while maintaining sensitivity to periodic patterns. Moreover, this approach demonstrates robustness to phase shifts and timing variations in the signal, a crucial advantage in real-world operating conditions where exact synchronization may not be guaranteed. These characteristics complement DNN’s deep feature learning capabilities particularly well, enabling the network to identify and learn from the most relevant temporal patterns for accurate fault classification.

The input layer of the DNN has twenty-one nodes, matching the number of dimensions in the input data pertaining to faults. The hidden layers have five subsequent layers consisting of 32, 64, 128, 64, and 32 neurons, respectively. Each layer uses the rectified linear unit (ReLU) activation function as depicted in Figure 5. The output layer consists of two neurons corresponding to the normal and fault classes. In the output layer, we used the softmax activation function, which offers probability distributions for precise fault classification. Adam is an optimization technique that may be used to update dense neural network weights iteratively based on training data, replacing the traditional stochastic gradient descent approach [33]. We used cross-entropy as the loss function to evaluate the accuracy measure. We stopped training if the loss metric did not improve after two consecutive epochs, preventing an overfitting curve. Thus, the proposed DNN is specifically designed to improve fault classification skills and leverages the model principles to automatically identify complex patterns from the input data.

## 3. Feature Extraction

This section presents our approach to statistical feature extraction for fault diagnosis. We employed eleven statistical features that effectively characterized the distribution and patterns in vibration data. The extracted features included mean, standard deviation, quartile medians (Q1, Q2, and Q3), minimum and maximum (peak-to-peak), kurtosis, skewness, root mean square (RMS), and energy. For parameter optimization, we utilized kurtosis as an advanced input feature, measuring signal sharpness to produce optimized features. The detailed optimization process using kurtosis is discussed in [34]. These optimization techniques enable comprehensive assessment of the data structure, particularly in identifying peaked, flat, or directionally biased distributions. Skewness quantifies distribution symmetry, with zero indicating normal or symmetric distributions, while kurtosis measures the relative heaviness of distribution tails compared to normal distributions. Both metrics provide crucial statistical characteristics for fault detection. The mean and standard deviation describe the central tendency and data spread, respectively, with variance and standard deviation being particularly useful for measuring data distribution during feature extraction [35]. Our methodology applies these statistical features to differentiate between expected (normal) and unusual (imbalance) patterns in time-domain data. The dataset comprises 2550 feature windows, with 70% allocated for training and 30% for testing. Each window spans 132 samples and is advanced with 25% overlap, generating multiple statistical features that characterize the vibration patterns under diverse operating conditions. The mathematical formulations for all statistical features are presented in Table 4.

## 4. Feature Signal Analysis for Fault Detection

The MAFAULDA dataset contains vibration data collected through industrial IMI sensors (601A01 and 604B31 accelerometers), which were positioned on the MFS to capture vibrations in radial, axial, and tangential directions. The data acquisition system included a Monarch Instrument MT-190 tachometer, Shure SM81 microphone, and National Instruments NI-9234 data acquisition modules operating at a 51.2 kHz sampling rate. The analysis extracted eleven fundamental features from the raw vibration data: mean, standard deviation, minimum, maximum, kurtosis, skewness, root mean square (RMS), energy, and median values (25%, 50%, 75%). The SVM and KNN methods applied these features to both raw and processed data, allowing the algorithms to learn and adapt to underlying patterns for effective classification. The DNN implementation uniquely incorporated autocorrelation analysis of accelerometer data, computed through FFT-based convolution, which captured temporal patterns and recurring signal characteristics crucial for fault detection.

**Figure 6:**
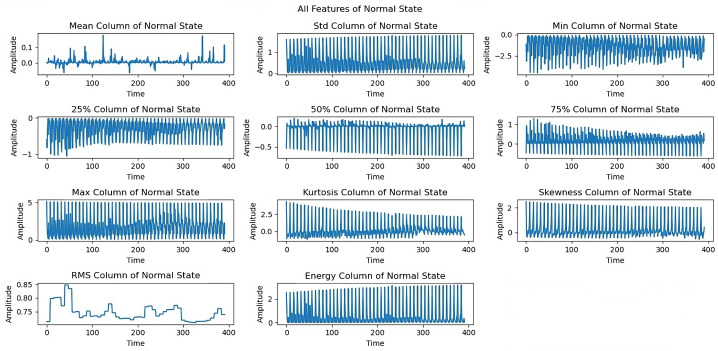
Analysis of 11 features in normal rotational sequences (737 rpm to 3686 rpm).

The dataset comprises vibration sequences at fixed rotation speeds from 254 to 3686 rpm, with approximately 60 rpm increments. These sequences were sampled at 50 kHz and analyzed using a sliding window approach. Windows of 132 samples were advanced with 25% overlap between consecutive windows, corresponding to a step size of 99 samples, resulting in 2550 feature windows spanning approximately five seconds of data. The 60 rpm increments were carefully chosen to provide comprehensive coverage of operational speeds while maintaining manageable data volume. The imbalance fault simulations were conducted using loads ranging from 6 g to 35 g. Under normal operation, the rotational frequency was consistently maintained for each load value below 30 g. These operating conditions maintained a constant speed within each sequence, allowing for reliable fault signature identification. Figure 6 shows the analysis of extracted features under normal rotational speeds. Loads equal to or exceeding 30 g limited the system’s ability to achieve rotational frequencies above 3300 rpm, revealing important characteristics for fault detection. The progressive load conditions (6 g to 35 g) reveal important system dynamics that are crucial for practical fault detection implementations.

**Figure 7:**
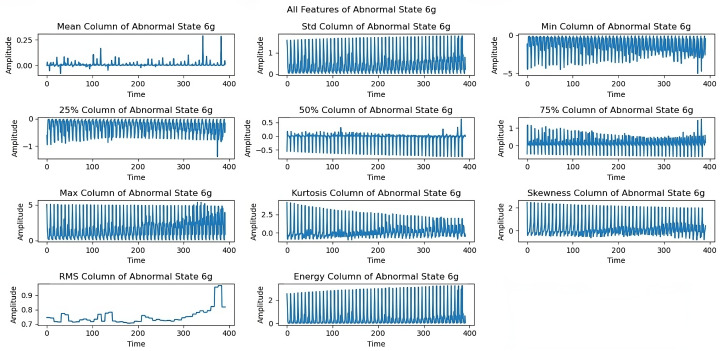
Feature analysis under 6 g imbalance: consistently maintained rotations.

**Figure 8:**
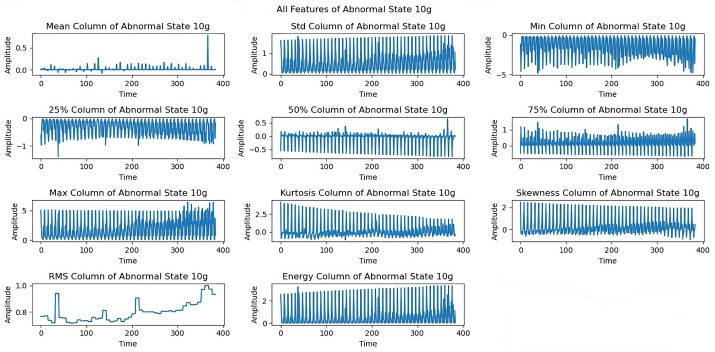
Feature analysis under the 10 g imbalance: stable rotational characteristics.

Different imbalance conditions show distinct behavioral patterns. At the 6 g imbalance (Figure 7), the mean, standard deviation, and kurtosis properties remained stable despite slight perturbations. The 10 g imbalance analysis (Figure 8) shows sustained rotational properties. Skewness and RMS features demonstrated characteristic changes in the system’s vibrational behavior under an increased imbalance load.

**Figure 9:**
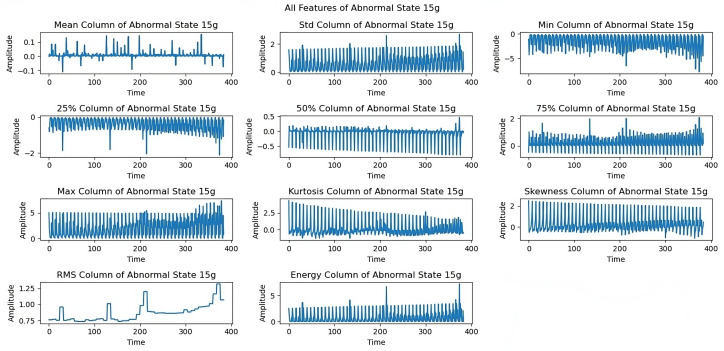
Feature analysis under the 15 g imbalance: assessing rotational stability.

**Figure 10:**
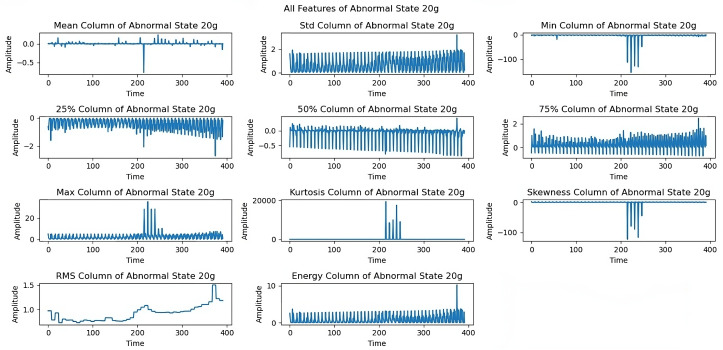
Feature analysis under the 20 g imbalance: dynamical variations.

**Figure 11:**
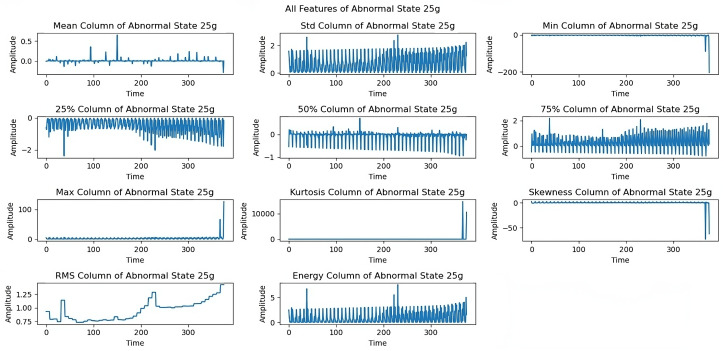
Feature analysis under the 25 g imbalance: investigating system resilience.

At the 15 g imbalance (Figure 9), variations in maximum, energy, and median parameters show how the system responded to the increased load. The 20 g load imbalance (Figure 10) created significant dynamical variations across all features, especially in skewness and kurtosis. The 25 g imbalance analysis (Figure 11) reveals the system’s response to substantial imbalances through standard deviation and minimum trends.

The analysis is expanded to a 15 g load imbalance as shown in Figure 9. Changes in important parameters, such as the maximum, energy, and median, provide a detailed analysis of how the system reacts to more extreme imbalance situations. The maximum value directly indicates peak vibration amplitudes, which typically increase under severe imbalance conditions. This parameter is crucial as it reveals potential threshold violations that could indicate severe mechanical stress on the system. The energy value, calculated as the sum of squared signal amplitudes provides a comprehensive measure of the overall vibrational intensity. Under extreme imbalance conditions, energy values show significant increases reflecting the higher vibrational content across the entire signal duration. This makes energy a sensitive indicator of severe imbalance conditions.

Figure 10 shows the effects of a 20 g load imbalance, resulting in dynamical variations in all features. Skewness and kurtosis are two characteristics that might show significant fluctuations, suggesting that the system is sensitive to increasing imbalance levels. In Figure 11, variations in all extracted features are represented under a 25 g imbalance. Similarly, trends in standard deviation and minimum provide insight into the system’s capability to manage important imbalance issues.

**Figure 12:**
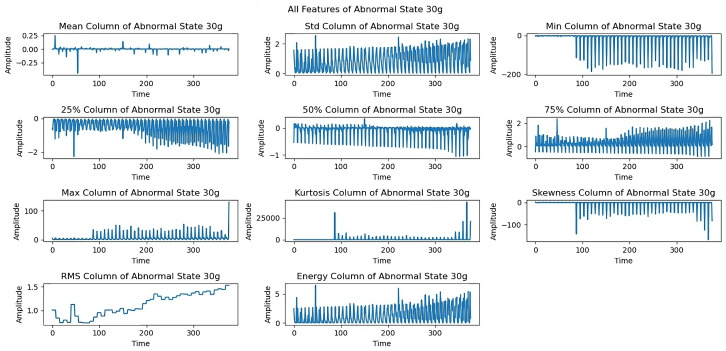
Feature analysis under a 30 g imbalance: limitations on rotational frequencies.

**Figure 13:**
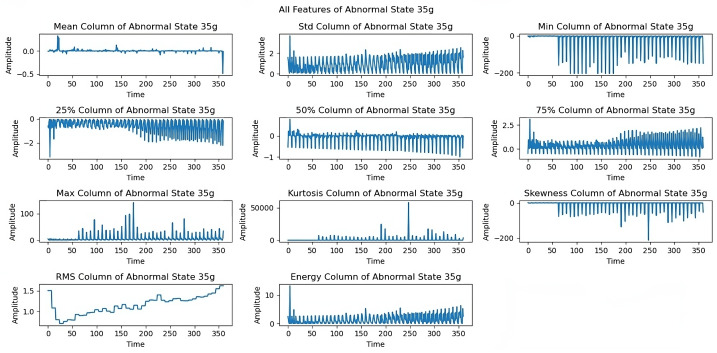
Feature analysis under a 35 g imbalance.

Figure 12 shows limitations to the feature performance by analyzing the effects of higher load imbalance. The 30 g load analysis reveals constrained rotational frequencies, limiting frequencies above 3300 rpm, and variations in maximum and energy features, indicating system stability issues when subjected to high loads. The dynamics of all extracted features under a severe load of a 35 g imbalance are examined in the last exploration as shown in Figure 13. At a 35 g load imbalance, significant changes appear across all features, particularly in mean, skewness, and RMS values, showing system behavior at operational limits. Each sequence spans five seconds at a 50 kHz sampling rate, providing detailed data for fault detection. In Figure 6, Figure 7, Figure 8, Figure 9, Figure 10, Figure 11, Figure 12 and Figure 13, the time is taken in microseconds.

## 5. Practical Consideration of SVM, KNN, and DNN Algorithms

### 5.1. Implementation Framework

Our implementation leverages Python’s robust machine learning libraries. For classification tasks, we utilized scikit-learn’s support vector classifier (SVC) for the SVM implementation and KNeighborsClassifier for KNN. The framework incorporates GridSearchCV for hyperparameter optimization through a systematic grid search, enhancing both SVM and KNN algorithms. To address dataset imbalance, we employed RandomOverSampler from the imbalanced-learn library. The implementation also relies on essential Python libraries: matplotlib.pyplot for visualization, pandas for data manipulation, NumPy for numerical operations, and SciPy for statistical measures like kurtosis and skewness. Feature standardization was achieved using scikit-learn’s StandardScaler, ensuring zero mean and unit variance for input features.

### 5.2. Performance Metrics

Our evaluation framework employs standard metrics to assess model performance. These metrics provide detailed insights into how well the models handle true positives (TPs), true negatives (TNs), false positives (FPs), and false negatives (FNs). The ROC curves, shown in Figure 3 and Figure 4, illustrate the trade-off between sensitivity and specificity across classification thresholds. The key metrics include the following: Accuracy, which involves measuring overall classification performance as expressed in Equation (3),

(3) $$\mathrm{Accuracy}=\frac{TP+TN}{TP+FN+TN+FP},$$

Precision is calculated by Equation (4), which is crucial when false positives are costly,

(4) $$\mathrm{Precision}=\frac{TP}{TP+FP},$$

Recall is obtained by Equation (5), which is essential when false negatives must be minimized,

(5) $$\mathrm{Recall}=\frac{TP}{TP+FN},$$

and F1 score, which provides a balanced measure of precision and recall is computed by using the following Equation:

(6) $$F1\;\mathrm{score}=\frac{2\times (\mathrm{Precision}\times \mathrm{Recall})}{\mathrm{Precision}+\mathrm{Recall}}$$

### 5.3. Comparative Analysis

Our experimental results demonstrate the effectiveness of optimization and oversampling strategies across all implemented algorithms. As shown in Table 5, the SVM implementation showed consistent improvement through optimization, with the baseline model achieving 87% in training and 85.9% in testing accuracy. The optimized version improved to 93% in training and 90.4% in testing accuracy, while the addition of oversampling further enhanced performance to 97.5% in training and 95.4% in testing accuracy. The KNN implementation followed a similar trajectory of improvement. The baseline model achieved 94% in training and 87.4% in testing accuracy; with optimization, it maintained 95% training accuracy and improved testing accuracy to 89.8%. The oversampled version demonstrated the best performance, reaching 95.7% in training and 92.8% in testing accuracy.

Most notably, our DNN implementation with autocorrelation features, computed using FFT-based convolution, showed exceptional performance. While the direct time-domain analysis achieved respectable results (97% in training, 95% in testing accuracy), the FFT-based autocorrelation features approach significantly improved performance to 99.8% in training and 99.7% in testing accuracy. These results underscore the importance of both optimization strategies and appropriate data preprocessing in fault diagnostics. The consistent improvement across all algorithms, particularly with oversampling and FFT-based autocorrelation feature analysis, suggests robust potential for real-world applications in predictive maintenance systems.

## 6. Illustration of Experimental Results

### 6.1. SVM

**Figure 14:**
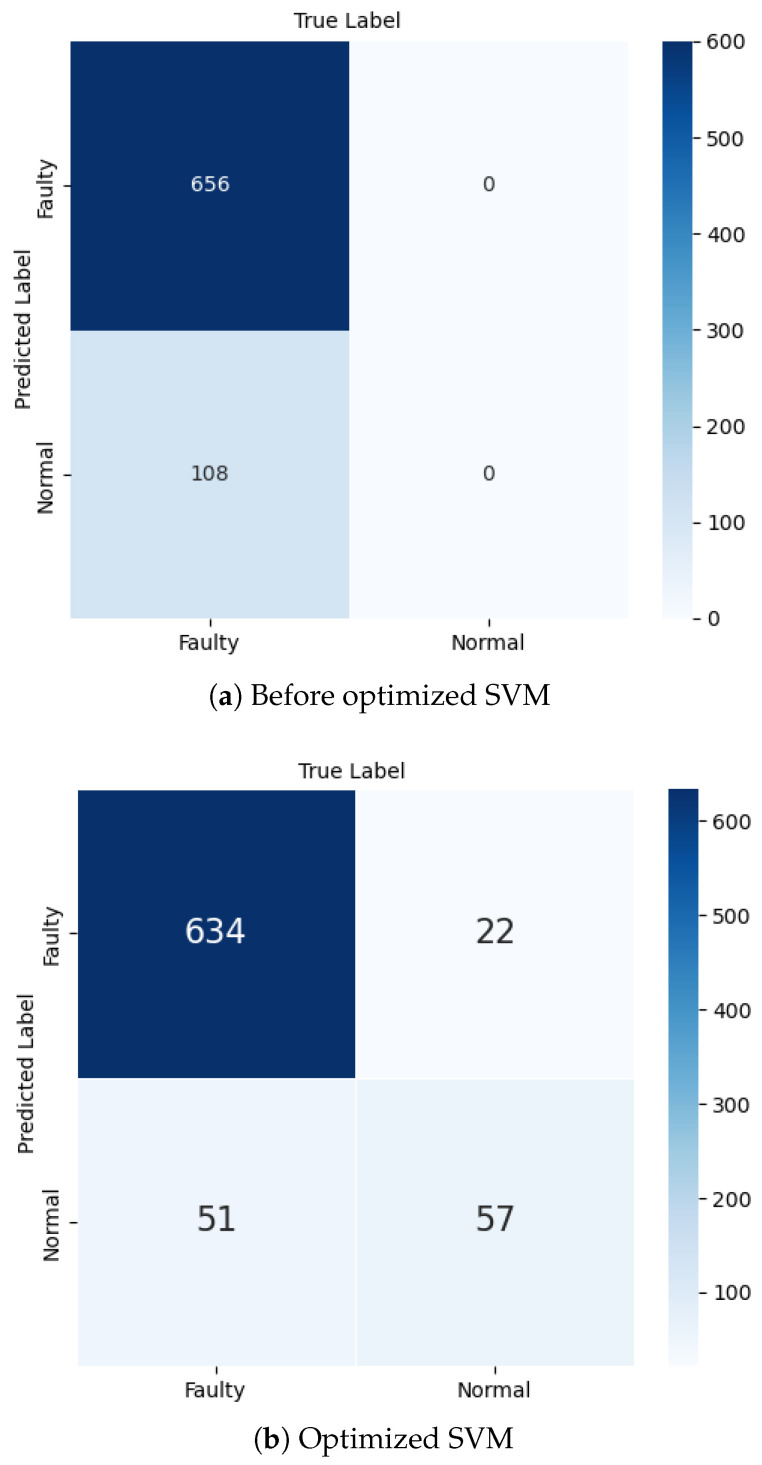

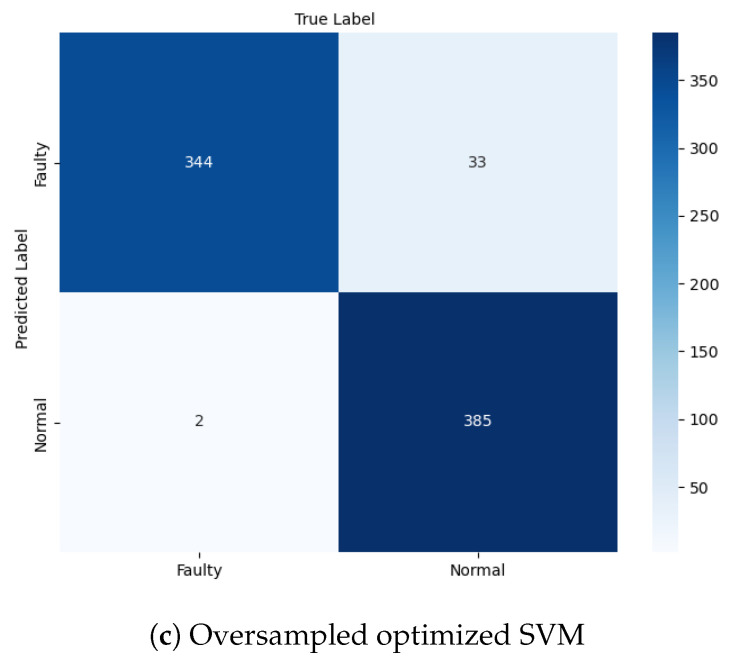
Faulty and normal classification using SVM.

Our experimental validation employed the MAFAULDA dataset [22] to thoroughly evaluate model performance. The classification results are best understood through confusion matrices, presented in Figure 14, which provide a comprehensive view of how well our models distinguish between normal and fault conditions. The detailed performance metrics derived from Figure 14a–c are summarized in Table 6. Among our implementations, the oversampled optimized SVM demonstrated remarkable performance, correctly identifying 344 faulty cases and 385 normal cases, while minimizing misclassifications to just 33 false positives and 2 false negatives. This translated to an impressive 95.4% accuracy, with the model achieving 91.2% precision and 99.4% recall. While the optimized SVM also showed strong performance with 97.2% precision, the unoptimized linear SVM, despite perfect precision (1.00), fell short in overall effectiveness with an accuracy of 85.8%, correctly identifying 656 positive cases but also recording 108 false negatives.

### 6.2. KNN

**Figure 15:**
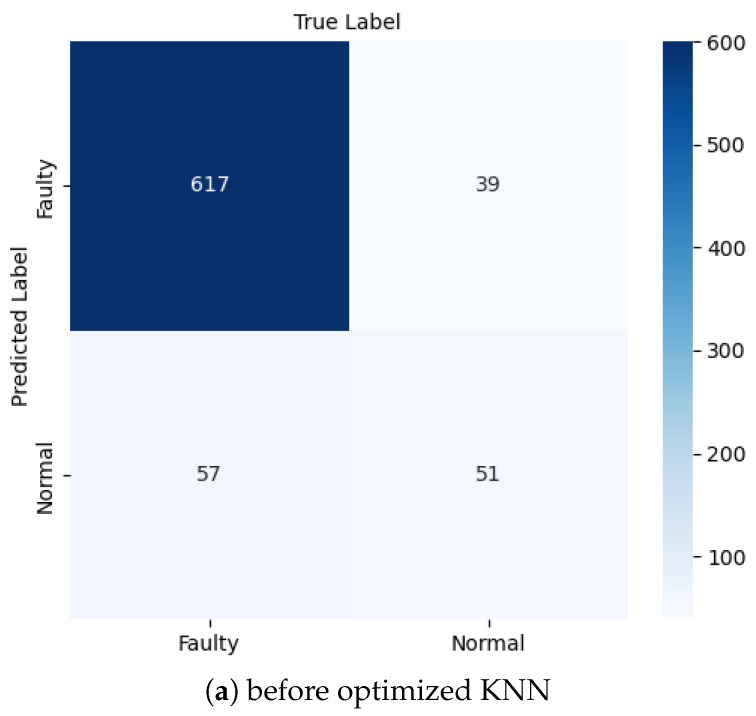

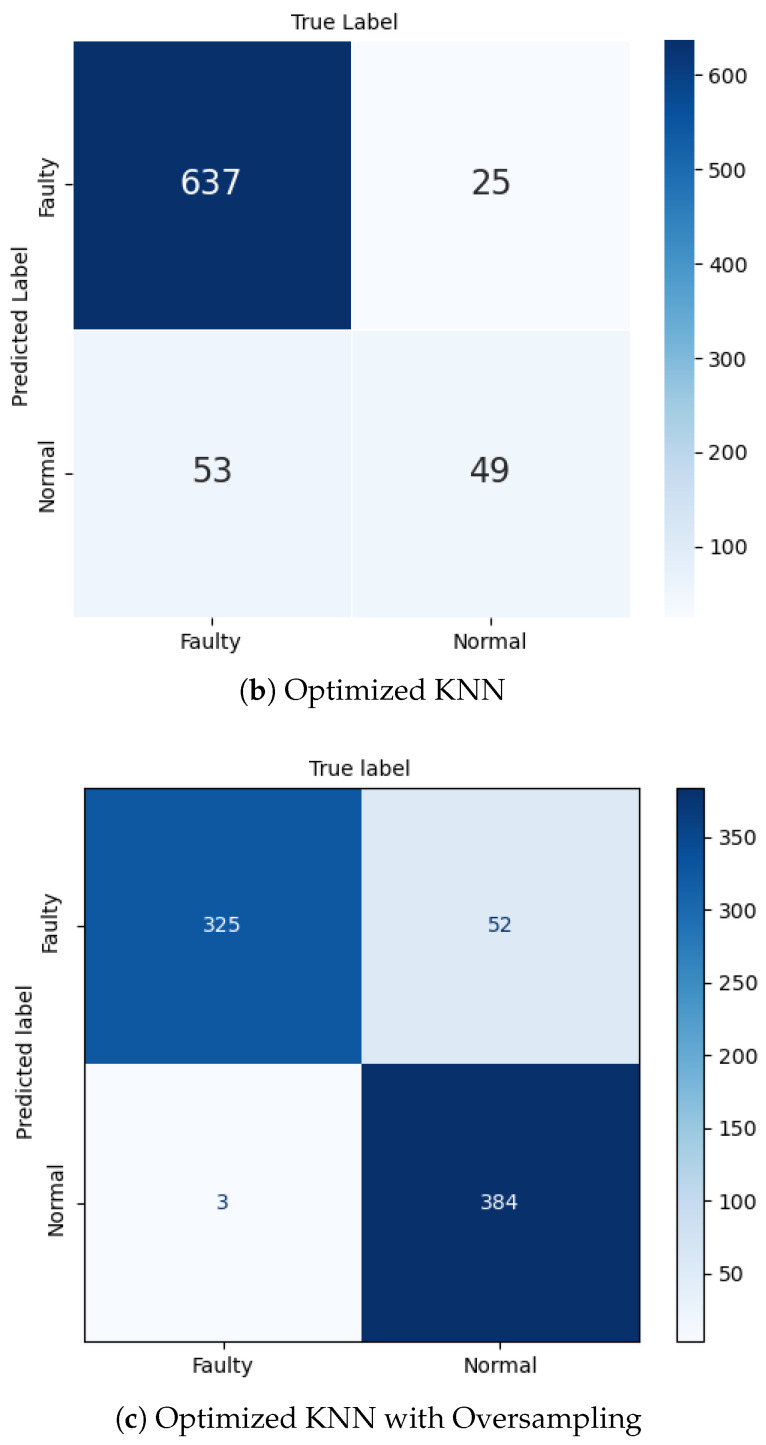
Faulty and normal classification using KNN.

Our KNN implementation underwent similar rigorous testing, with results visualized in Figure 15. The oversampled optimized variant proved most effective, successfully identifying 325 faulty and 384 normal cases, although it produced 52 false positives and 3 false negatives. Table 7 presents the complete performance metrics, calculated using Equations (3)–(6). The oversampled approach achieved 92.8% accuracy with an exceptional 99.1% recall, while the base-optimized KNN showed strong discrimination capabilities with 637 correct positive identifications. The unoptimized version, while achieving a respectable 87.4% accuracy, demonstrated room for improvement through our optimization strategies.

### 6.3. DNN

**Figure 16:**
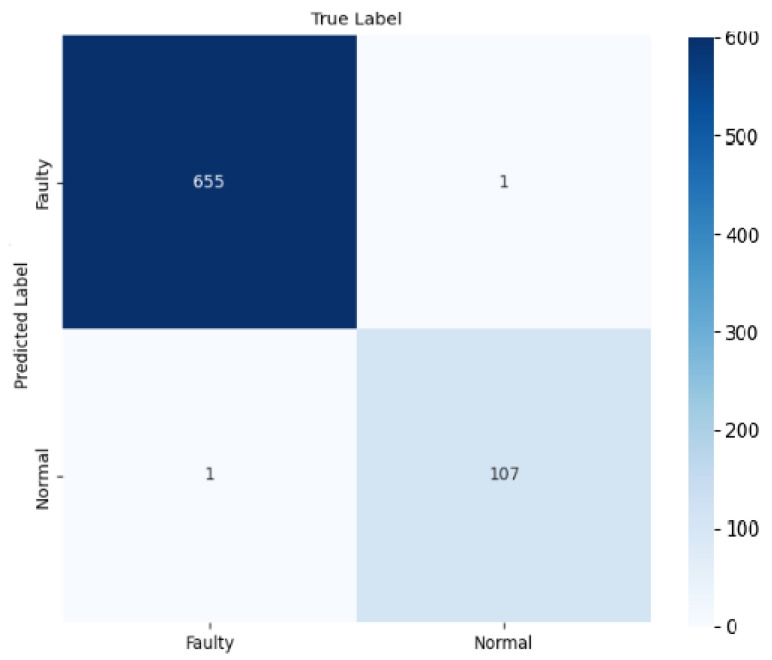
Faulty and normal classification using DNN.

In developing DNN architecture using the dense NN method, we leveraged FFT-based autocorrelation features to enhance the model’s ability to recognize fault patterns. This approach processes raw vibration data through FFT-based autocorrelation analysis, where fault signatures become more distinguishable through the enhancement of temporal patterns and periodic relationships in the signal. As demonstrated in Figure 16, this transformation significantly improved classification performance. By effectively filtering noise and focusing on relevant frequency components, our DNN achieved an outstanding 99.7% accuracy, compared to 95% in time-domain analysis as shown in Table 5.

Based on the FFT-based autocorrelation fed data, DNN successfully identified 655 faulty and 107 normal cases, producing 01 false positive and 01 false negative. The significance of the performance becomes clear when considering that the dataset was not balanced through oversampling. This approach achieved a remarkable accuracy of 99.7% with an exceptional 99.9% precision and 99.8% recall. The confusion matrix is depicted in Figure 16.

## 7. Conclusions

This paper introduces effective methods for predicting induction motor faults, aiming to minimize losses and prevent disasters through advanced fault detection techniques. By leveraging SVM, KNN, and DNN, we precisely classify motor states as either “normal” or “imbalance”. Our findings demonstrate significant improvements over previous studies, notably with accuracy for optimized oversampled SVM reaching 95.4% and optimized oversampled KNN achieving an accuracy of 92.8%. These methods, enhanced by optimization and oversampling strategies, contribute significantly to fault detection in induction motors. The best-performing algorithm was DNN with FFT-based implementation of autocorrelation, achieving an impressive 99.7% accuracy. This aligns with trends in technology-driven fault identification and offers substantial benefits for industrial processes such as increased efficiency, reduced downtime, and enhanced safety

In future work, the model’s robustness will be enhanced by testing it on the testbed under varying load conditions and real-world noise. The training dataset will also be expanded with diverse fault scenarios, and adaptive learning techniques will be incorporated to improve the generalization and reliability of the model. 

## Figures and Tables

**Table 1:** Machinery fault simulator (MFS) specifications.

Parameter	Value
Motor & 1/4 CV DC	
Frequency Range	700–3600 rpm
System Weight	22 kg
Axis Diameter	16 mm
Rotor	15.24 cm
Bearings Distance	390 mm
**Data Acquisition System**	
Accelerometers	Three IMI 601A01 model
	One IMI 604B31 model
Tachometer	Monarch MT-190
Microphone sensor	Shure SM81
Data Acquisition Modules	Two NI 9234
Sample Rate	51.2 kHz
**Sampling Parameter**	**Value**
Sequence Duration	5 s
Sampling Rate	50 kHz
Samples per Sequence	250,000
**Sequence Types**	
Normal Sequences	Rotation speed: 737 to 3686 rpm
Imbalance Faults	Load values: 6 g to 35 g

**Table 2:** Hyperparameter grid for optimized SVM.

Hyperparameter	Values
*C*	0.1, 1, 10, 100
γ	0.01, 0.1, 1, 10

**Table 3:** Hyperparameters used in optimized KNN.

Hyperparameter	Values
n_neighbors	3, 5, 7, 9
*p*	1 (Manhattan), 2 (Euclidean)

**Table 4:** Statistical formulas of features.

Feature	Formula
Mean	$\bar{x}=\frac{1}{N}\sum_{i=1}^{N}\left(x_{i}\right)$
Standard Deviation	$\sigma =\sqrt{\frac{1}{N}(\sum_{i=1}^{N}{\left(x_{i}-\bar{x}\right)}^{2})}$
Minimum	$\min(x_{i})$
First Quartile (Q1)	$\left(Q1=\frac{1}{4}\left((N+1)\mathrm{th}\right)\right)$
Median (Q2)	$\left(Q2=\frac{1}{2}\left((N+1)\mathrm{th}\right)\right)$
Third Quartile (Q3)	$\left(Q3=\frac{3}{4}\left((N+1)\mathrm{th}\right)\right)$
Maximum	$\max(x_{i})$
Kurtosis	$\frac{\sum_{i=1}^{N}{\left(x_{i}-\bar{x}\right)}^{4}}{\left(N-1\right)\sigma^{4}}$
Skewness	$\frac{\sum_{i=1}^{N}{\left(x_{i}-\bar{x}\right)}^{3}}{\left(N-1\right)\sigma^{3}}$
Root Mean Square (RMS)	$\sqrt{\frac{1}{N}\left(\sum_{i=1}^{N}{\left(x_{i}\right)}^{2}\right)}$
Energy	$\sqrt{\sum_{i=1}^{N}{\left|x_{i}\right|}^{2}}$

**Table 5:** Comparison of algorithms.

Algorithm	Training	Testing
	Accuracy	Accuracy
Unoptimized SVM	87%	85.9%
Optimized SVM	93%	90.4%
Oversampled optimized SVM	97.5%	95.4%
Unoptimized KNN	94%	87.4%
Optimized KNN	95%	89.8%
Oversampled optimized KNN	95.7%	92.8%
Time-domain based DNN	97%	95%
FFT based DNN	99.8%	99.7%

**Table 6:** Calculation of accuracy, precision, recall, and F1 score of the model using SVM.

Algorithm	TP	FP	FN	TN	Accuracy	Precision	Recall	F1 Score
Linear SVM	656	0	108	0	0.86	1.00	0.86	0.92
Optimized SVM	634	22	51	57	0.90	0.97	0.93	0.95
Oversampled optimized SVM	344	33	2	385	0.95	0.91	0.99	0.95

**Table 7:** Calculation of accuracy, precision, recall, and F1 score of the model using KNN.

Algorithm	TP	FP	FN	TN	Accuracy	Precision	Recall	F1 Score
Unoptimized KNN	617	39	57	51	0.87	0.94	0.92	0.93
Optimized KNN	637	25	53	49	0.90	0.96	0.92	0.94
Oversampled optimized KNN	325	52	3	384	0.93	0.86	0.99	0.92

## Data Availability

Available on the public repository website: See reference [24].
